# A Review of Energy Supply for Biomachine Hybrid Robots

**DOI:** 10.34133/cbsystems.0053

**Published:** 2023-09-26

**Authors:** Zhiyun Ma, Jieliang Zhao, Li Yu, Mengdan Yan, Lulu Liang, Xiangbing Wu, Mengdi Xu, Wenzhong Wang, Shaoze Yan

**Affiliations:** ^1^ School of Mechanical Engineering, Beijing Institute of Technology, Beijing 100081, P. R. China.; ^2^ Department of Mechanical Engineering, Carnegie Mellon University, Pittsburgh, PA 15213, USA.; ^3^ Department of Mechanical Engineering, Tsinghua University, Beijing 100084, P. R. China.

## Abstract

Biomachine hybrid robots have been proposed for important scenarios, such as wilderness rescue, ecological monitoring, and hazardous area surveying. The energy supply unit used to power the control backpack carried by these robots determines their future development and practical application. Current energy supply devices for control backpacks are mainly chemical batteries. To achieve self-powered devices, researchers have developed solar energy, bioenergy, biothermal energy, and biovibration energy harvesters. This review provides an overview of research in the development of chemical batteries and self-powered devices for biomachine hybrid robots. Various batteries for different biocarriers and the entry points for the design of self-powered devices are outlined in detail. Finally, an overview of the future challenges and possible directions for the development of energy supply devices used to biomachine hybrid robots is provided.

## Introduction

Biomachine hybrid robots (BHRs) use biomechanical interface technology to establish a connection between the external information system and animals to control their movement [1,2]. In contrast to conventional bionic robots, they are free of complex mechanical structures, and due to the direct adoption of the animal body, they have superior moving characteristics and long-lasting range. In particular, with the development of integrated circuit (IC) technology, researchers have proposed the use of BHRs in important scenarios, such as urban and wilderness rescue operations [3–5], environmental monitoring [6,7], and hazardous area survey [8,9].

Currently, the study of BHRs in terms of movement control is based on the electrical stimulation of the nerves or muscles of organisms [10–12]. Researchers have already reported BHRs based on insects, such as cockroaches [13,14], beetles [15–18], moths [19,20], locusts [21], and other animals such as pigeons [22,23], rats [8,24,25], and fish [26,27]. Although the BHRs do not require external energy to maintain their motion, the signals used to control the biological vehicle are normally output through the control backpack carried by the robots. The backpack incorporates a control output chip and several low-power electronic components used to implement scenario-specific tasks [4,12,28]. To accomplish long mission endurance, the energy supply of the control backpack must be considered. As different biological carriers have different requirements for the energy equipment, BHRs' power supply is an important issue.

**Fig. 1.**
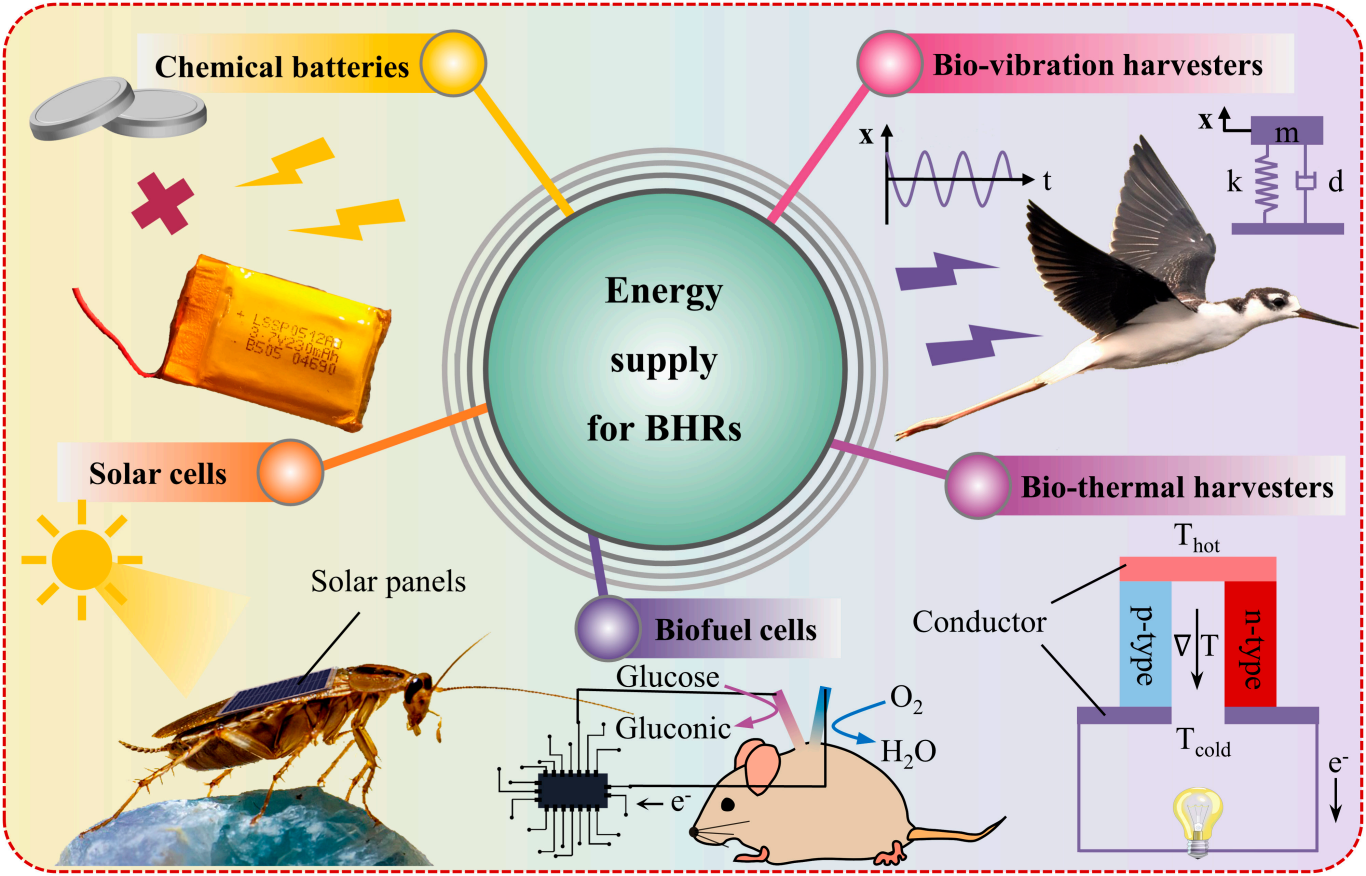
An overview of the energy supply for biomachine hybrid robots.

The energy supply method of BHRs is shown in Fig. 1. Chemical batteries are common energy suppliers that have a relatively stable energy output and are widely used in BHRs. However, chemical batteries need frequent replacement or recharging, which severely limits the lifetime of the BHRs and can even cause damage to the vitality of the biocarrier [29]. Moreover, batteries with high energy density are also generally large in mass and volume, accounting for nearly 80% of the weight and volume of the entire backpack [30]. This considerably impacts the movement of low-load capacity organisms, such as insects. As a result, certain researchers are focusing on solar cells [31,32], biofuel cells [7,33], biothermal energy harvesters [34,35], and biovibration energy harvesters [36–38] for BHRs. They aim to replace the original chemical batteries and achieve self-powering of the electronic components carried by the BHRs.

As an essential aspect of BHR research, energy supply directly determines the future development of BHRs. However, to the knowledge of the authors, no review of current research exists on energy supply in BHRs. This paper presents a comprehensive overview of the various energy supply methods in BHR research, from the selection of chemical batteries for different biocarriers to the development and application of various energy harvesters. Finally, insights on the main problems encountered in BHRs' energy supply and the future development direction are also presented based on existing research, aiming to promote further development of energy supply devices for BHRs.

## Chemical Batteries

After a long period of development, chemical batteries have had a profound impact on modern society [39]. As the most direct form of energy supply, chemical batteries are also the first choice for powering the control system and electronic components of BHRs. This section summarizes some of the options available in existing BHR studies. Additionally, the relevant content in terms of both insect carriers and other biological carriers is presented, focusing on the technical features in the selection of energy supply cells for the different carriers.

### Invertebrate control backpack energy supply batteries

Insects are extensively distributed and exist in large numbers, making them the most abundant group of invertebrates on Earth. Due to their superior movement range, including flying, crawling, and jumping [40], there has been an increasing amount of research on insect-based BHRs in recent years. With all researchers using chemical batteries to power the backpack of BHRs, battery selection for the insect–machine hybrid robots is also an issue worthy of discussion. As different insects vary in size and load capacity, the physical and energetic parameters need to be balanced when selecting the battery, which is to best match the particular insect carrier.

The Malagasy cockroach is a common insect that has been used in multiple studies as a research vehicle for BHRs because of its large size and high loading capacity. In terms of the choice of batteries to power them, from the first American cockroach–machine hybrid robot developed by Holzer and Shimoyama [41] in 1997 to subsequent studies by Sato and colleagues [42,43], they used a Li-ion battery weighing approximately 2 g to power the control backpack. Li and colleagues [14,44] used a coin battery to power components such as low-power Bluetooth wireless transceivers on the control backpack, which was experimentally tested to work 12 h for each component.

**Fig. 2.**
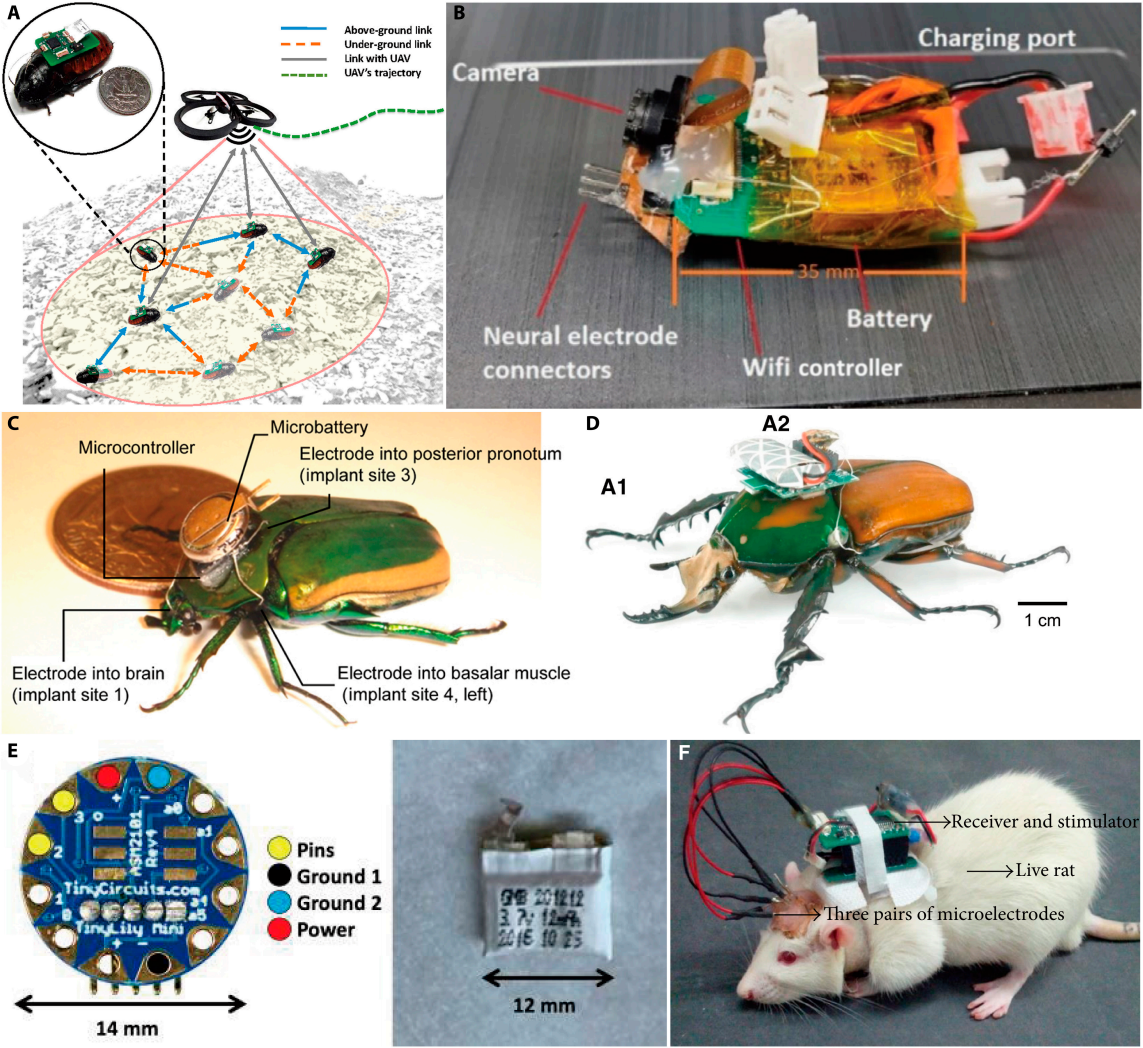
BHRs using chemical batteries for energy supply. (A) Cockroach–machine hybrid robot for disaster rescue and other scenarios [9]. Copyright 2017 Elsevier. (B) CameraRoach with a camera system [4]. Adapted from [4] with the permission under the terms of the CC license. (C and D) Beetle–machine hybrid robot powered with a coin battery and micro-Li-ion battery, respectively [50,51]. (C) Copyright 2009 Sato et al. (D) Copyright 2015 Elsevier. (E) Li-Po battery powering the jellyfish swim controller [56]. Adapted from [56] with the permission under the terms of the CC-BY license. (F) Li-Po battery powering the control backpacks for a rat [8]. Adapted from [8] with the permission under the terms of the CC license.

Li-Po batteries have been favored in subsequent studies due to their high energy density and lightweight. Bozkurt and colleagues [3,9,45,46] have chosen Li-Po batteries to power the cockroach–machine hybrid robot they developed for disaster rescue and other scenarios (Fig. 2A). To keep the weight of the backpack well below the payload capacity of the cockroach [3], the chosen batteries ranged from 20 to 90 mAh. In their experimental conditions, the robot could operate for 1 h when powered by a 50 mAh Li-Po battery [45]. Rasakatla et al. [4] used a 7.4 V, 125 mAh Li-Po battery to power a cockroach–machine hybrid robot called CameraRoach (Fig. 2B). The robot with on-board camera feedback and can be navigated by remote control and a boost converter was used to achieve power supply for 30 min or longer. In addition, Ma and colleagues [21,47] developed a microcontrolled backpack for controlling locust jumps, which was powered by an 8 mAh coin battery and weighed only 0.95 g. A fully charged battery can support the backpack for more than 10 min and trigger locust jumps more than 20 times.

Compared to cockroaches and locusts, some flying insects, such as beetles and moths, are more sensitive to additional weight. Therefore, when selecting the battery, in addition to meeting the basic electrical requirements, it is also necessary to consider the basic parameters of the entire battery, such as weight and size, to ensure that the carrier insect can fly properly. Bozkurt et al. [48] made a moth–machine hybrid robot by inserting a microsystem in the pupa stage of the insect, with the control system powered upfront by two 8 mAh coin batteries that weighed 120 mg. On this basis, they used a Li-Po battery (3.6 V, 8.5 mAh) weighing only 300 mg later to power the control backpack [49], which could operate for more than 5 h with continuous pulses. Sato et al. [50] have been working on the beetle–machine hybrid robot, using a battery to power the control backpack that is constantly evolving. In 2009, they used a rechargeable Li-ion coin cell with a 3.4 mAh capacity and a 160 mg weight to power the microcontroller (Fig. 2C). They later used an 8.5 mAh, 350 mg rechargeable micro-Li-ion battery to power the backpack (Fig. 2D), bringing the overall mass of the backpack down to 1.26 g [51]. Later, they used a 3.7 V, 10 mAh Li-Po micro-battery to power the backpack [17,52], reducing the total mass of the backpack to 1.2 g, which is lower than the payload capacity of the beetles [53]. Recent studies have also used a rechargeable LiTiO_2_ battery to power the control backpack of the beetle–machine hybrid robot, which weighs just 200 mg [54,55].

Apart from insects, Xu et al. [56–58] developed a portable, self-contained microelectronic controller for controlling the motion of jellyfish, which was powered by a 10 mAh Li-Po battery (Fig. 2E). A comparative analysis of the parameters in these studies shown in Table 1 reveals that the batteries used for invertebrate are dominated by coin batteries, micro-Li-ion, and Li-Po batteries, which are relatively small in size and weight. The mass of these batteries generally ranges from 0.1 to 0.4 g, which reflects the importance of the mass parameter in the choice of batteries for invertebrates.

**Table 1.** List of reported energy supply types for BHRs with chemical batteries.

Year	Carrier animal	Battery type	Capacity/mA·h	Weight/g	Voltage/V	Working hours/h	Reference
1997	Cockroach	Li-ion battery	—	—	3	—	[41]
2022, 2023	Cockroach	Li-ion battery	120	2.5	—	—	[42,43]
2016	Cockroach	Li-Po battery	20–90	0.8–2.5	—	—	[3]
2017	Cockroach	Li-Po battery	50	—	—	1	[45]
2022	Cockroach	Li-ion battery	125	—	7.4	>0.5	[4]
2023	Cockroach	Li-Po battery	50	1.4	—	0.83	[13]
2016, 2017	Cockroach	Coin battery	140	1.9	3	12	[14,44]
2022, 2023	Locust	Li-Po battery	8	0.71	—	—	[21,47]
2007	Hawkmoth	Coin battery	8	0.12	—	—	[48]
2009	Hawkmoth	Li-Po battery	8.5	0.3	3.6	>5	[49]
2018	Beetle	Coin battery	8	—	1.55	—	[150]
2009	Beetle	Coin battery	3.4	0.16	3	—	[50]
		Li-ion battery	8.5	0.35	3.9	—	
2018	Beetle	Li-ion battery	8.5	0.35	3.7	—	[151]
2023	Beetle	Li-ion battery	8	0.2	1.5	—	[54,55]
2015	Beetle	Li-ion micro-battery	8.5	0.35	3.9	—	[51]
2022	Beetle	Li-Po battery	20	0.403	3.7	—	[152]
2016, 2022	Beetle	Li-Po micro-battery	10	0.35	3.7	—	[17,52]
2016	Rat	Lithium cells	120	—	3.7	8	[8,24,25]
2016	Turtle	Li-Po battery	2,600	86.5	—	—	[62]
2015	Pigeon	Polymer battery	120	—	3.7	—	[23,60,61]
2009	Fish	—	—	—	3	—	[26]
2020, 2021	Jellyfish	Li-Po cell	10	—	—	—	[56–58]

### Vertebrate control backpack energy supply batteries

Besides invertebrates, many researchers have also developed control systems for vertebrates, such as rats, fish, and pigeons. The selection of batteries carried by these animals focuses more on their energy properties as there are no more load capacity limitations. Comparison of related studies (Table 1) shows that most researchers have chosen Li-Po batteries with better performance in all aspects. A more representative example is the rat–machine hybrid robot developed by Zheng and colleagues [8,24,25,59], which employs two 120 mAh Li-Po batteries to power a miniature camera and electronic components carried by the rat, enabling the animal to complete the maze escape task (Fig. 2F). Yang and colleagues [23,60,61] developed a navigation control system for pigeons, powered by a 3.7 V, 120 mAh Li-Po battery. Wireless controllers for controlling goldfish and carp were developed by Kobayashi et al. [26] and Peng et al. [27], respectively, who used a 3 V lithium battery to power the controllers. Kim et al. [62] developed a controller mounted on the head of a turtle that relies on a human brain–computer interface to control remotely its movements. The controller is powered by a 2,600 mAh Li-Po battery, and its total weight is 171.5 g, of which the battery weighs 86.5 g.

## Study of Self-Powered Devices

Most of the published literature uses batteries to power control backpacks. Even though the size of the batteries becomes smaller and smaller, the need for high energy density remains a requirement and they remain the majority of the payload of the control backpack [63]. Thus, some researchers have started to develop electrical energy conversion devices that can effectively harvest different forms of energy from the surrounding environment or the animal itself, which could achieve self-powering of the BHRs. This chapter contains a summary overview of current research on self-powered devices for BHRs.

### Solar cells

As a widely distributed natural source of clean energy, solar energy has been used for various purposes such as solar power generation [64], solar thermal utilization [65], photochemical reactions [66], and photobiological applications [67]. Continuous technological advances have decreased the cost of solar power [68], and it is already possible to achieve energy conversion of power densities of 10 mW/cm^2^ or higher in outdoor lighting conditions [69]. In recent years, with the development of microelectromechanical system (MEMS) processing and molding technology [70,71], new solar cells, such as organic solar cells [72], chalcogenide solar cells [73], and quantum solar cells [74], have emerged, in addition to the original silicon solar cells. Due to their smaller substrate and passivation thickness, it is possible to achieve a balance in device weight and power output, which also sets the stage for applications in the energy supply of BHRs.

**Fig. 3.**
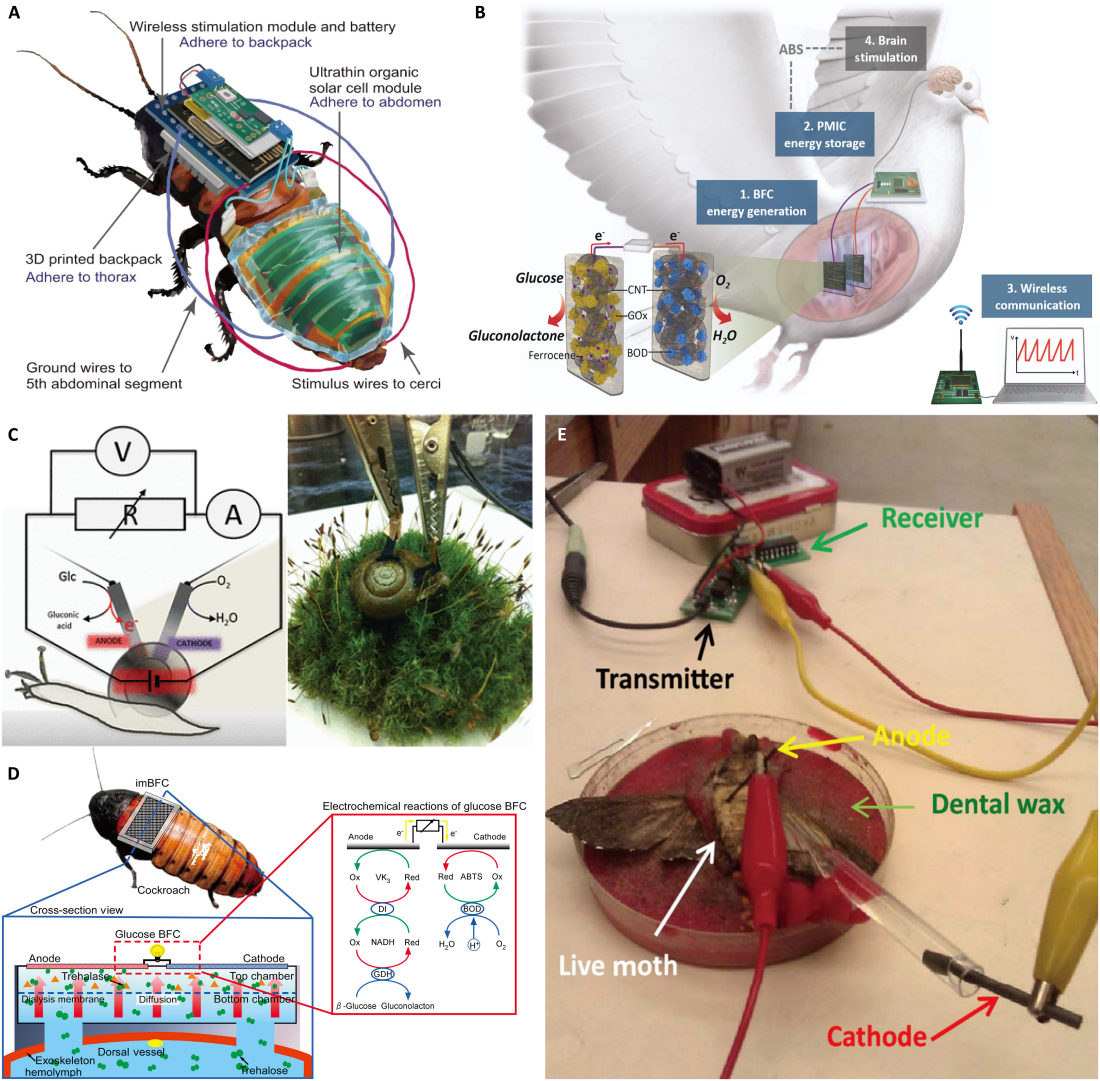
BHRs using solar and biofuel cells for energy supply. (A) Ultrasoft organic solar cell integrated on the body of a cockroach–machine hybrid robot [31]. Adapted from [31] with the permission under the terms of the CC-BY license. (B) Biofuel cell to power the pigeon–machine hybrid robot [33]. Copyright 2021 Elsevier. (C) Biofuel cell implanted in a snail [90]. Copyright 2012 American Chemical Society. (D) Enzymatic biofuel cell to power a cockroach control backpack [7]. Copyright 2016 Elsevier. (E) Biofuel cell implanted in a moth [92]. Adapted from [92] with the permission under the terms of the CC-BY license.

Solar cells have already been used to power BHRs. Reissman and colleagues [75,76] used a SOIC packaged solar chip with an area of 25 mm^2^ and a mass of 63 mg to power a moth–machine hybrid robot, which operated very similarly to a battery at 4 V. In natural sunlight, solar cells can produce a steady output of nearly 200 μW, but the power may only be 4 μW under artificial lighting conditions or shade. Bozkurt and colleagues [32,77] report a self-powered device based on solar energy, which was used to power a control backpack and recharge the battery carried by a cockroach–machine hybrid robot (Fig. 3A). This automatic charging operation was achieved by designing a virtual fence in which the insects could be brought near a light source and kept in the area during battery charging. Experimental studies have shown that a 20 mA battery can be fully charged within 2 h under direct and indirect sunlight, a focused white light-emitting diode, or an incandescent lamp at a distance of 8 cm. Under bright light conditions, the solar cell can reach the fastest charging speed, which can be fully charged in 10 to 30 min. Kakei et al. [31] reported an ultrasoft organic solar cell module integrated into a cockroach–machine hybrid robot (Fig. 3A). They used a combination of ultrathin film electronics and a bonded–nonbonded interleaved structure to avoid compromising the basic movement of the cockroach. This body-hugging, ultrathin organic solar cell achieves a power output of 17.2 mW.

Solar cells can achieve continuous power supply for BHR control backpacks, but their power generation is affected by the environment and the weather in which the biocarriers operate. In particular, the energy-harvesting effect is often insufficient to cover the basic needs of some of the more light-sheltered biocarriers.

### Biofuel cells

The energy conversion devices that convert the chemical energy of molecules in living organisms into electrical energy are called biofuel cells [78]. Compared to conventional batteries, the concentration of reactants in biofuel cells is constantly replenished by body fluids, which allows the bioenergy conversion device to work continuously. Thus, it is possible to use biofuel cells to avoid the external charging of devices or batteries [79]. Moreover, biofuel cells can theoretically operate indefinitely, as long as there is a continuous supply of fuel in the organism [80]. Biofuel cells mainly consist of microbial fuel cells and enzyme fuel cells [81,82]. Among them, enzyme biofuel cells (EBFCs) use enzymes as catalysts to oxidize glucose in vivo and are considered to be the most suitable implantable bioenergy harvesters.

The continuous development of biofuel cell technology has enabled EBFCs to be successfully implanted into animals such as rats [83–85], rabbits [86,87], lobsters [88], tilapia [89], and pigeons [33]. Cosnier and colleagues [84,85] presented the first functional implantable glucose biofuel cell (GBFC) implanted into the retroperitoneal gap of freely moving rats (Fig. 3C). The GBFC produced a peak specific power of 24.4 μW/ml, which outperforms to current pacemaker specifications. Martin and colleagues [86] implanted a bioelectronic device of an EBFC into rabbits and monitored its function in vivo for 2 months. They used a remote transmission system to allow the EBFC to be charged and discharged in vivo, charging a 100 kΩ load for 30 min per day. The EBFC delivered 16 μW/ml of power for 16 days until 2 months later, when the output was reduced because of inflammation. Similarly, Miyake et al. [87] inserted a glucose-oxidized needle anode into a vessel in the ear of the rabbit and achieved a power of 0.42 μW at the 0.56 V voltage state. Katz and colleagues [88] implanted an EBFC into lobsters, and in experimental tests, the open-circuit voltage and short-circuit current were about 600 mV and 1 mA, respectively. At the optimum resistance of 500 Ω, the maximum power generated was around 0.16 mW, and the power density was around 0.64 mW/cm^2^. Lee et al. [33] reported on the implantation of a biofuel cell into a pigeon–machine hybrid robot that carries a brain stimulator (Fig. 3B). The study showed that based on glucose and oxygen in the pigeon, the power of the biofuel cell was 0.12 mW in vitro and 0.08 mW in vivo. By using a power management integrated circuit, the in vivo energy could be harvested at a rate of 28.4 mJ for more than 10 min, sufficient for intermittent neurostimulation.

Compared to the research mentioned above, implanting biocatalytic electrodes to harvest energy from smaller species presents higher difficulty. In contrast, with the advancement of microminiature processing technology, many researchers have also investigated small biofuel cells, mainly in aquatic animals, such as snails [90] and clams [91], and in insect carriers such as moths [92] and cockroaches [6,7,93]. Katz and his team implanted a biofuel cell into snails [90] and clams [91], with an open circuit voltage of 530 mV and a short circuit current of 42.5 μA, respectively (Fig. 3C). The maximum power was 7.45 μW generated by the biofuel cells at an optimum resistance of 20 kΩ, and the power density was around 30 μW/cm^2^. The open circuit voltage and short circuit current within the clams were approximately 300 to 400 mV and 30 to 100 μA, respectively. The biofuel cell produced a maximum power of 10 μW and a power density of 40 μW/cm^2^ at the optimum resistance of 3 kΩ.

Rasmussen et al. [93] designed a biofuel cell with a dual enzyme alglucosidase/glucose oxidase-alginate anode, a bilirubin oxidase oxygen cathode as the electrode, and an Os complex as the electronic relay, which grafted onto the polymer backbone. The biofuel cell was implanted into female *Blaberus discoidalis* through an abdominal incision. The power density of the biofuel cell reached approximately 55 μW/cm^2^ at 0.2 V, with a reduction of only 5% at 2.5 h after surgery. Shoji et al. [6,7] reported an enzymatic biofuel cell that can supply energy to a cockroach control backpack (Fig. 3D). The biofuel cell generated electrical energy from algal sugars in the hemolymph of the cockroach via an alginate and glucose dehydrogenase reaction system that dehydrogenated the β-glucose obtained by hydrolysis of algal sugars. The performance of the biofuel cell was evaluated by obtaining a maximum power output of 333 μW at 0.5 V and a power density of 285 μW/cm^2^. The same experiment was successfully conducted by Schwefel et al. [92], who implanted a biofuel cell into a moth (Fig. 3E). Additionally, they produced a cockroach (*Gromphadorhina portentosa*) that was mounted with biofuel cells and a self-powered moth–machine hybrid robot for environmental monitoring.

While biofuel cells have demonstrated considerable value in self-powering electronic devices in living organisms, their practical application remains relatively challenging due to their short life span and low power density. Currently, the use of nanomaterials in biofuel cells has been favored by researchers and it has been proven to be an important way to improve the efficiency of biofuel cells [94,95]. In addition, the biocompatibility of materials for biofuel cell electrodes has been investigated to ensure their safety and stability in living organisms [96–98]. This research will push the application of biofuel cells for self-powered BHRs.

### Biothermal energy harvesters

The harvesting of biothermal energy is achieved by a thermoelectric conversion device, which converts biological heat into electrical energy based on the Seebeck effect [99,100]. The basic principle of this effect can be expressed as

$$V=\alpha N\left(T_{h}-T_{c}\right)$$ (1)

$$P=\frac{V^{2}}{R_{g}}=\frac{\alpha^{2}N^{2}{\left(T_{h}-T_{c}\right)}^{2}}{R_{g}}$$ (2)

where *V* is the potential generated by the conversion, *P* is the corresponding power, *α* is the Seebeck factor, *N* is the number of thermocouples, (*T*_h_ − *T*_c_) is the temperature gradient between the hot and cold ends of the generator, and *R*_g_ is the internal resistance of the thermoelectric generator.

Currently, the main research in biothermal energy harvesting is still focused on the harvesters of human thermal energy. To achieve self-powered wearable electronic devices for humans, a lot of research has been conducted on thermoelectric conversion devices for harvesting human heat [101–103]. Based on the need for wearability, various aspects have been investigated including thermal design for external temperature differences [104], cold-side/hot-side thermal resistance optimization [105], mechanical design for optimizing deformability at the material and device level [106,107], and optimization from flexibility to extension and structural design from two-dimensional to three-dimensional features [108,109]. In addition, a thermoelectric energy harvester mounted on the neck of a sheep has been reported, with a maximum average output power of 173 μW [35].

**Fig. 4.**
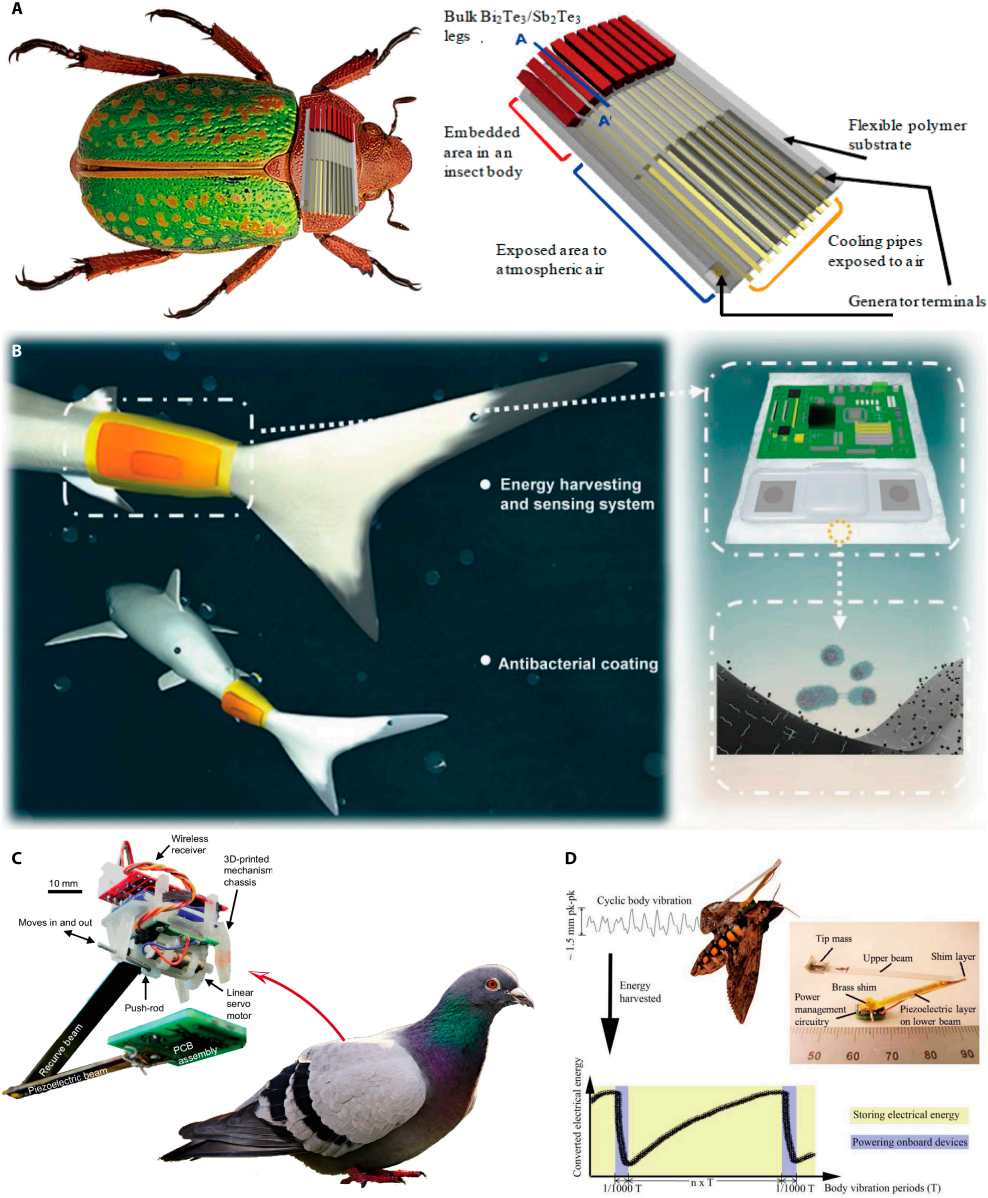
The study of energy supply for BHRs through harvesting biothermal and biovibration energy. (A) Thermoelectric energy harvester for beetles [34]. Copyright 2008 IEEE. (B) Airbag tribology nanogenerator with antibacterial coating for fish [124]. Copyright 2022 John Wiley and Sons. (C) Piezoelectric energy harvester for birds [127]. Copyright 2013 ASME. (D) Piezoelectric energy harvester mounted on the thorax of a moth [131]. Copyright 2011 SPIE.

In terms of research on thermoelectric energy supply devices for BHRs, Ghafouri et al. [34] designed an implantable micro-thermoelectric energy harvester for beetles (Fig. 4A). The selected generator thermocouple material to be placed on the back of the beetle during its pupal stage was Bi_2_Te_3_/Sb_2_Te_3_. The cold end of the harvester was exposed to the air to create a temperature difference, and the thermocouple and cold end were adhered to a flexible polymer substrate. The harvester achieved an output power density of 10 μW/cm^2^ at a temperature difference of 11 °C.

Thermoelectric energy harvesters for biothermal energy conversion have proven to be one of the better ways of self-powered supply for BHRs, due to their high energy conversion efficiency and environmental adaptability. However, because of the limitations of the operating principle of thermoelectric energy harvesters, a sufficient temperature difference must be present to produce the more desirable electrical energy. As a result, harvesters often have a relatively low power density, making it difficult to match the higher power supply requirements. In recent years, researchers have worked on various aspects of these harvesters, such as the design and synthesis of organic, inorganic, and hybrid materials [110] and the design of the micro-nano structures on the device surface [111]. Research has focused on thermoelectric energy harvesters with higher conversion efficiency and power density. As a result, thermoelectric energy conversion methods based on organisms will have very promising applications in future biosensing systems and the energy supply for BHRs.

### Biovibration energy harvesters

Biological vibrations, a type of motion prevalent in nature, are not greatly affected by their environment [112]. With the development of materials science and engineering technology, researchers are dedicated to developing advanced energy harvesters to efficiently convert biological vibration energy into electrical energy. For vibration-electric conversion mechanisms, three of the most well-investigated methods are based on piezoelectric, electromagnetic, and electrostatic conversions [113], all of which have important applications in the harvesting of vibrational energy from living organisms.

To summarize recent research, some important applications of biovibration energy harvesters have been investigated, such as pacemakers [114,115], wearable electronic devices [116,117], and smart pasture monitoring [118–120]. Many scholars have designed vibration energy harvesters based on the movements of aquatic organisms like fish [121–125] and wild animals like pigeons, pheasants, and giraffes [36,37,126–129] for the self-energy supply of electronic components such as the biomonitors they are carrying. Wang et al. [124] developed an airbag tribological nanogenerator covered with an antibacterial coating to power a wearable monitoring system carried by fish (Fig. 4B). The nanogenerator produced a peak power of 0.74 mW per oscillation of the fishtail, and its voltage reflected the behavior of the fishtail at real time. Shafer et al. [37,127] determined the frequency of steady flight by measuring the acceleration of a flying bird. On this basis, they used the maximum output power of a recently developed piezoelectric energy harvester to estimate the power that could be obtained from one flying bird. They proved the feasibility of collecting vibrational energy harvesting from flying birds and bats to power microcontrollers and biomonitoring electronics (Fig. 4C). Nakada et al. [129] reported an electromagnetic generator that could be placed beneath the skin or in the abdominal cavity of a bird, which was tested and found to produce an average power of 0.47 mW with a magnetic field length of 2 mm and a coil length of 15 mm. This output is sufficient for the power supply of electronic devices carried by birds.

The research of vibrational energy harvesters for small animals, such as insects, is more challenging than for larger animals. With the development of microfabrication technology, some researchers have also investigated vibrational energy harvesters for small insects, such as moths [130–132], beetles [38], and bees [133,134]. Chang [130] used an electromagnetic vibrating energy harvester to convert the energy produced by the vibration of the wings of *Manduca sexta* into electrical energy. The resonant energy-harvesting device was fixed on the thorax of the moth, and the energy harvester had a total weight of 1.28 g and could produce 1 mW of power at 1 V. Reissman and colleagues [131] designed a piezoelectric energy harvester installed on the thorax of a moth, while the rectifier circuit was designed so that the generated energy was first stored in a capacitor and then distributed to power the carrier electronics (Fig. 4D). In free-flight tests, the energy harvester generated up to 59 μW of electrical energy, with a root mean square (RMS) power of 35 μW for instantaneous power generation. In 2011, Aktakka et al. [38] reported a piezoelectric vibration energy harvester used to convert vibrations generated by the root of the *Cotinis nitida* sheath wing into electrical energy. The piezoelectric material used in the device was a double-layer lead zirconate titanate (PZT), which was machined into a spiral shape by femtosecond laser technology. The center of the two spirals was fixed on the thorax in front of the beetle's two elytra and the end touched the elytra, which were subjected to lifting pressure during the vibration process. The total mass of the device was less than 200 mg, and the average volume of the individual spirals was 10.85 mm^3^. The energy harvester output at least 45 μW of electrical power. The design of the spiral form allows for energy harvesting over a wide range of frequencies to ensure effective winging energy conversion in the presence of different beetles and flight attitudes.

Self-powered devices based on biovibration energy harvesting have been employed for key research applications in the supply of low-power components carried by living organisms, due to their relatively simple design and adaptability. This has provided a good basis for the development of self-powered devices for semimechanical hybrid robots and also for the development of self-powered devices for BHRs. Analysis of current research shows that current vibration energy harvesters still face limitations, such as low energy conversion efficiency and power density, narrow frequency bands, and insufficient miniaturization and integration, which challenges their actual promotion and application on small carriers.

To enhance the output of vibration energy-harvesting devices and broaden their operating frequency band, various aspects have been investigated, such as composite technology [135], hybrid energy conversion mechanisms [136], multi-stable structures [137], and frequency up-conversion structures [138]. With the development of MEMS technology, researchers have also conducted numerous studies on the miniaturization and integration of vibration energy harvesters [139,140]. The results of these studies also confirm that vibration energy harvesters have an important future application in the supply of energy to control backpacks for BHRs.

## Future Challenges and Possible Approaches

As new conceptual robots, BHRs have important application prospects in future scenarios, such as animal monitoring and wildlife rescue. The energy supply system directly determines the practical application of BHRs. The current research on energy supply systems for BHRs is still in its infancy. Based on our practical research and conclusions, the development of energy supply devices for BHRs can be further advanced in the future by addressing the following challenges and implementing appropriate approaches.

### High energy density energy supply devices

Whether they are chemical batteries or self-powered energy harvesters, high energy density is an important direction for future development. In the case of BHRs, a sufficient supply of energy can practically promote their application in different scenarios. The increase in energy density will also promote the reduction of the weight of the entire energy supply device for the same power requirements, which is critical for BHRs with smaller load capacities. With the creation of new materials and advances in micro-nano technology, the main directions for enhancing energy density are provided in terms of composite materials and microscale structural design. Recently, many studies have reported on electrode materials with high energy density [141], tiny chemical cell designs [142], and cases of enhancing the density of harvester devices from the aspect of material and micro-nano technologies [143–145].

### Biocompatibility of energy supply devices

Certain implantable energy supply devices require special attention to the biocompatibility of the materials involved. Considering the complexity of the physiological activity of the biocarriers of BHRs, the implanted self-powered devices cannot affect the basic movement of the BHRs. In addition, any device touching the skin or tissues must be nonbiotoxic to avoid serious immune reactions that could affect its lifetime. Research on biocompatible materials for biofuel cell electrodes [97] and biothermal and vibration energy harvesters [146,147] has rapidly increased in recent years.

### Compound energy supply

The output performance of the energy supply device determines the effective lifetime of a BHR. Since the various energy harvesters have their advantages and limitations, composite energy supply technologies have attracted the attention of researchers. By reasonably harvesting multiple environmental energy sources and applying multiple energy conversion mechanisms, the space utilization efficiency of energy supply devices can be improved effectively and the power output can be also increased. Many researchers have investigated composite energy harvesters from various aspects in recent years, such as materials, structural design, and system integration [148].

### Stability of energy supply

Long-term stability of the energy supply system is a basic requirement for the effective control of BHRs. Especially for energy conversion supply methods, it is difficult to ensure that a long-term stable energy supply can be achieved, due to its dependence on the energy conversion source. Many researchers have used in this context energy management circuits [149]. The storage of the converted energy by integrated microcapacitors or batteries and the supply of energy to the BHRs ensure a stable output to the control backpack.

### Environmentally friendly energy supply

In the application of BHRs, situations such as accidental deaths are unavoidable. As BHRs are used in the natural environment, addressing the impact of energy supply systems on the environment is a vital challenge. Therefore, it is important to investigate environmentally friendly energy supply devices for BHRs based on material science and technology in the future.

## Conclusions

The energy supply system of biohybrid robots (BHRs) has received a great deal of attention from researchers, as it is a vital part of their future development and practical application. At present, the energy supply of BHRs is still dominated by chemical batteries, which have a stable output. The urgent need for lower weight, smaller size, and high energy output density is reflected in the choice of chemical batteries (Table 1). A common problem is how to achieve longevity of energy supply and overcome the impact on the lifetime of BHRs, due to the need for battery replacement or recharging. To achieve that, self-powered devices have been investigated in four areas: solar, bioenergy, biothermal, and biovibration energy (Table 2). The current studies on energy harvesters are characterized by low conversion efficiency and output power, unstable energy supply, and poor biocarrier compatibility. These shortcomings are major concerns. Based on the current status of energy supply for BHRs, it is clear that the development of high energy density energy supply devices, biocompatibility, composite energy harvesting, and stability of energy supply will be important challenges for future research. The development of new materials, the design of micro- and nano-structures, and the development of composite energy supply devices will be important ways to address these limitations.

**Table 2.** Summary of research on self-powered devices for BHRs.

Year	Carrier animal	Energy source	Power/μW	Weight/mg	Voltage/V	Power density/μW·g^−1^	Power density/μW·cm^−2^	Reference
2008	Moth	Solar energy	200/4	63	4	3,174.6/63.5	—	[75]
2014	Cockroach	Solar energy	223e3	—	4.5	—	—	[77]
2022	Cockroach	Solar energy	17.2e3	—	1.92	15.9e6	—	[31]
2012	Cockroach	Bioenergy	—	—	0.2	—	55 ± 6	[93]
2016	Cockroach	Bioenergy	333	—	0.6	—	285	[6,7]
2019	Fish	Bioenergy	—	—	0.41	—	6.3	[89]
2021	Pigeon	Bioenergy	80	—	0.55	—	—	[33]
2012	Snail	Bioenergy	7.45	—	0.53	—	30	[90]
2012	Clams	Bioenergy	10	—	0.3–0.4	—	40	[91]
2013	Lobster	Bioenergy	160	—	0.6	—	640	[88]
2011	Rabbit	Bioenergy	0.42	—	0.56	—	260	[87]
2013	Rat	Bioenergy	6.5	—	0.275	—	—	[85]
2013	Rat	Bioenergy	0.35	—	0.14	—	0.175	[83]
2014	Moth	Bioenergy	0.12	<100	0.1	—	9	[92]
2008	Beetle	Biothermal	0.8	14.286	—	56	10	[34]
2022	Fish	Biovibration	740	—	225	—	—	[124]
2013	Pigeon	Biovibration	75–220	11.9e3	9–25	6.3–18.5	—	[127]
2010	Moth	Biovibration	1,000	1,280	1	781.3	—	[130]
2011	Moth	Biovibration	59	292	5.3	202	—	[131]
2011	Beetle	Biovibration	11.5	86.5	0.875	133	—	[38]
2021	Bee	Biovibration	3.6	62.4	0.3	57.7	—	[134]

## Data Availability

The data used to support the findings of this study are available from the corresponding author upon request.
